# Mitochondrial trafficking and redox/phosphorylation signaling supporting cell migration phenotypes

**DOI:** 10.3389/fmolb.2022.925755

**Published:** 2022-07-22

**Authors:** Nathaniel Shannon, Randi Gravelle, Brian Cunniff

**Affiliations:** ^1^ Department of Pathology and Laboratory Medicine, Redox Biology Program, University of Vermont Larner College of Medicine, Burlington, VT, United States; ^2^ University of Vermont Cancer Center, University of Vermont Larner College of Medicine, Burlington, VT, United States

**Keywords:** redox signaling, phosphorylation, cell migration, mitochondrial trafficking, focal adhesion (FA)

## Abstract

Regulation of cell signaling cascades is critical in making sure the response is activated spatially and for a desired duration. Cell signaling cascades are spatially and temporally controlled through local protein phosphorylation events which are determined by the activation of specific kinases and/or inactivation of phosphatases to elicit a complete and thorough response. For example, A-kinase-anchoring proteins (AKAPs) contribute to the local regulated activity protein kinase A (PKA). The activity of kinases and phosphatases can also be regulated through redox-dependent cysteine modifications that mediate the activity of these proteins. A primary example of this is the activation of the epidermal growth factor receptor (EGFR) and the inactivation of the phosphatase and tensin homologue (PTEN) phosphatase by reactive oxygen species (ROS). Therefore, the local redox environment must play a critical role in the timing and magnitude of these events. Mitochondria are a primary source of ROS and energy (ATP) that contributes to redox-dependent signaling and ATP-dependent phosphorylation events, respectively. The strategic positioning of mitochondria within cells contributes to intracellular gradients of ROS and ATP, which have been shown to correlate with changes to protein redox and phosphorylation status driving downstream cellular processes. In this review, we will discuss the relationship between subcellular mitochondrial positioning and intracellular ROS and ATP gradients that support dynamic oxidation and phosphorylation signaling and resulting cellular effects, specifically associated with cell migration signaling.

## Introduction

Two primary reversible post translational modifications, protein oxidation and phosphorylation, can elicit cooperative or divergent cell signaling responses affecting numerous cell processes including cell proliferation (Yao et al., 2019), cell migration (Hurd et al., 2012; Cao et al., 2015), transcription (Riedl and Egly, 2000; De Nigris et al., 2001; Al-Mehdi et al., 2012), stress response (Hamada et al., 2020), immune cell activation (Davidson et al., 2003; Gostner et al., 2013; Iwasaki et al., 2020) and more. These modifications directly impact protein structure and function, hence altering their downstream cell signaling cascades (Karasev et al., 2018; Fu et al., 2019). Mitochondria have emerged as an important source of ROS that contribute to redox signaling (Horn et al., 2017; Jezek et al., 2020) while being the primary source of cellular ATP required for cellular energy and protein phosphorylation. Mitochondria are dynamic organelles that vary in size, shape and location depending on cell type (normal and disease), energy status and metabolic demand for mitochondrial metabolites (Tilokani et al., 2018).

Mitochondria produce ROS and ATP at the electron transport chain (ETC) which takes place in the inner mitochondrial membrane (IMM). Electrons are passed from NADH and FADH_2_ through IMM bound protein complexes, with subsequent pumping of H^+^ ions to the intermembrane space (IMS). H^+^ ions are pumped from the IMS through the ATP synthase and into the mitochondrial matrix to generate ATP (Zhao et al., 2019). ROS generation occurs when the electrons from NADH/FADH_2_ leak out of the protein complex and bind with O_2_ to form superoxide (O_2_^−^) which can be enzymatically converted to H_2_O_2_ via the mitochondrial superoxide dismutase (SOD2) (Cadenas and Davies, 2000; Turrens, 2003). Approximately 0.2–2% of the electrons flowing through the ETC, under physiological conditions, can leak out to cause oxidation of proteins proximal to mitochondria (Cadenas and Davies, 2000; Turrens, 2003).

Not only can mitochondria produce ATP and ROS, but they can also regulate calcium (Ca^2+^) concentrations which also regulate mitochondrial function. A flux of mitochondrial Ca^2+^ causes activation of the dehydrogenases in the tricarboxylic acid (TCA) cycle, which are the rate limiting steps during oxidative phosphorylation; therefore, causing an increase in NADH which eventually feeds into the ETC (Duchen, 1992; Maechler and Wollheim, 2000; Rizzuto et al., 2000). The mitochondria can also associate with the endoplasmic reticulum (ER), which is involved in Ca^2+^ storage and release. Therefore, the interaction between mitochondria and the ER can lead to different Ca^2+^ associated pathways such as increased mitochondrial bioenergetics or even cell death (Carreras-Sureda et al., 2018; Marchi et al., 2018). The relationship between the mitochondria and Ca^2+^ signaling throughout the cell is extensive and not the central focus of this review.

The subcellular positioning of mitochondria, and the localized activity of mitochondria, drives intracellular gradients of ATP and ROS and therefore mitochondrial trafficking is necessary for localized accumulation of these molecules (Schuler et al., 2017; Alshaabi et al., 2021). A large body of research supports a key role for ROS-dependent redox signaling in regulating cell migration phenotypes (Hurd et al., 2012). Emerging research now shows the subcellular positioning of mitochondria also supports cell migration phenotypes (Desai et al., 2013; Altieri, 2017; Schuler et al., 2017), providing an interesting, yet unresolved, link between mitochondrial trafficking and redox signaling in cell migration. In this review we will discuss the relationship between mitochondrial positioning and the downstream signaling cascades elicited from localized mitochondrial ROS (mROS) and ATP with a focus on cell migration.

### Sources of ROS and redox signaling

**Figure 1:**
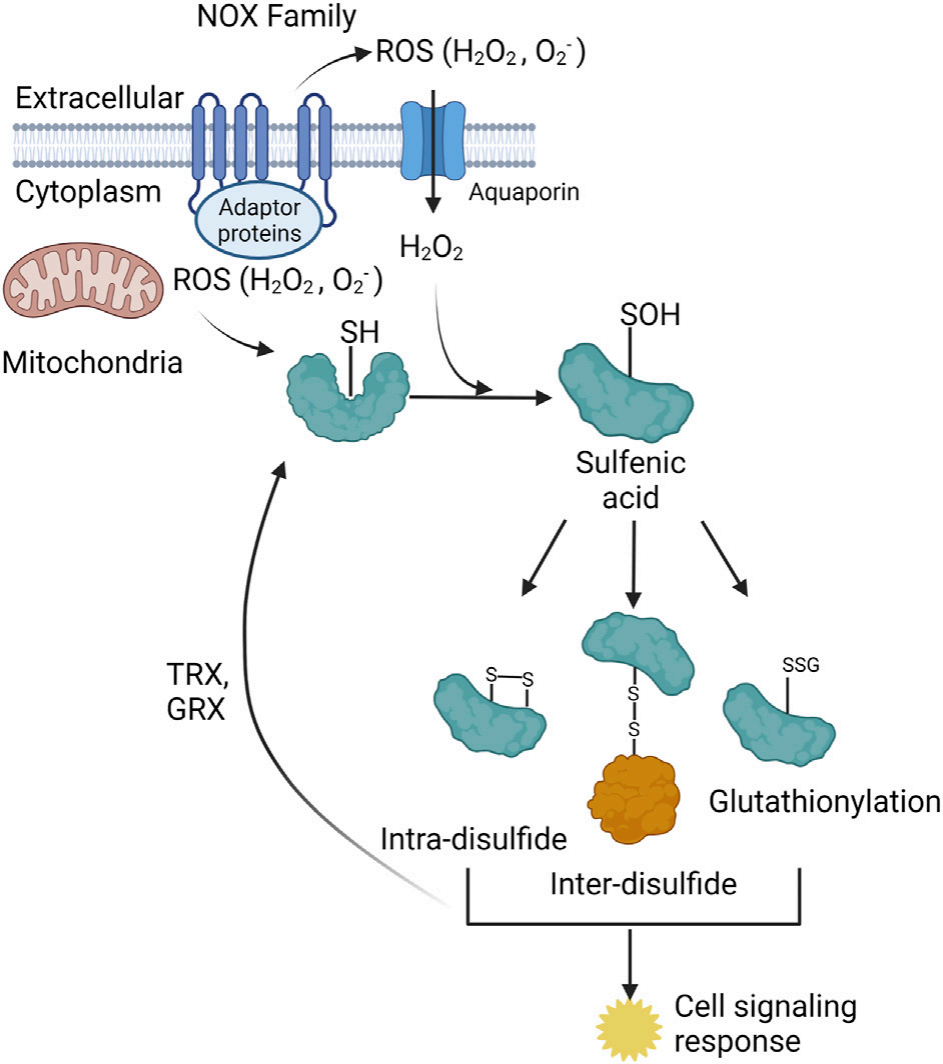
The two primary cellular sources of reactive oxygen species (ROS) are the NADPH oxidase (NOX) family of enzymes (NOX1-5, DUOX 1–2) and the mitochondrial electron transport chain (ETC). The NOX enzymes produces ROS (O_2_^-^ and H_2_O_2_) towards the extracellular space, O_2_^-^ is spontaneously or enzymatically (via SOD) dismutated to H_2_O_2_. H_2_O_2_is either transported through membrane channels or passed through the plasma membrane to elicit signaling in the cytoplasm. Mitochondria produce ROS into the mitochondrial matrix or the intermembrane space. Mitochondrial H_2_O_2_ can exit the mitochondria and signal in the cytoplasm. The ROS from both sources participate in redox-dependent signaling through oxidation of target cysteine residues on proteins. Cysteine sulfenic acids can form intra-disulfide bonds, inter-disulfide bonds, and become glutathionylated. All three of these species elicit several cell signaling responses within the cell. These three protein species can be converted back to the reduced thiol state via thioredoxin (TRX) and glutaredoxin (GRX) proteins.

The oxidation of target proteins occurs through ROS, specially hydrogen peroxide (H_2_O_2_) reacting with a free thiol (-SH) to form a sulfenic acid (-SOH), typically on cysteine residues, but can also react with methionine, tryptophan, and tyrosine residues (Hoshi and Heinemann, 2001; van der Vliet et al., 2018) (Figure 1). Chemical cell signaling events are well characterized for the reversible oxidation of cysteine residues (Berndt et al., 2007; Garcia-Santamarina et al., 2014); however, far less is understood about the role of oxidation of methionine, tryptophan, and tyrosine residues. Specific, structurally distinct and solvent accessible cysteine residues are targets for oxidation by H_2_O_2_, and these modifications result in structural and functional changes in target proteins (Cecarini et al., 2007) (Figure 1). Similar to protein phosphorylation/dephosphorylation cascades, the reversible oxidation of specific cysteine residues modulates signaling pathways that govern all facets of cell physiology (Yanes et al., 2010). Physiochemical characteristics of oxidized cysteine residues in target proteins underlie the specificity and hierarchy of responses in redox signaling. ROS, like H_2_O_2_, regulate cellular physiology through direct oxidation of cysteine residues in target proteins, or via inactivation of resident scavenger/chaperone proteins (Nguyen and Sok, 2003; Dustin et al., 2020). Redox signaling through “redox-relays” utilizes the H_2_O_2_ reactive peroxiredoxin (PRX) family of enzymes as intermediates for transferring oxidation to target proteins through inter-disulfide exchange (Sobotta et al., 2015; Stocker et al., 2018a; Stocker et al., 2018b; Kim and Jang, 2019). The reversible oxidation of proteins plays a central role in regulating cell signaling cascades that govern all facets of cellular responses (Holmstrom and Finkel, 2014).

Similar to oxidation, protein phosphorylation is a reversible post-translational modification that typically occurs on serine, threonine, and tyrosine residues (Rao et al., 2013). Phosphorylation of target proteins occurs when the gamma-phosphate of ATP is transferred to the hydroxyl group of an amino acid and this is accomplished by a set of proteins known as kinases (Endicott et al., 2012). The phosphate group can be removed by phosphatases, and this will return the residue back to the hydroxyl group, thus making this process reversible (Barford et al., 1998). In certain cell signaling cascades, these two reversible post translational modifications can converge to cooperatively promote signaling or compete to downregulate signaling (Chiarugi et al., 2003; Giannoni et al., 2005; Grintsevich et al., 2017; Londhe et al., 2020). Evolutionarily there are conserved cysteine residues proximal to a Ser/Thr/Tyr residue in various eukaryotic kinases that regulate activity, thus further demonstrating the dynamics between oxidation and phosphorylation (Byrne et al., 2020). A key example of this is the activation of kinases via oxidation of cysteine residues in the active site and the inactivation of protein tyrosine phosphatases via oxidation of active site cysteines (Ostman et al., 2011; Dustin et al., 2020), leading to prolonged phosphorylation of a target protein. A primary example is the oxidation of the epidermal growth factor receptor (EGFR) at Cys797 leading to enhanced tyrosine kinase activity (Paulsen et al., 2011). Inactivation of the phosphatase and tensin homologue (PTEN) phosphatase occurs during muscle differentiation when there is an increase in the oxidation of PTEN, leading to decreased activity which causes an upregulation of the PI3K/AKT/mTOR pathway since these target proteins are able to remain phosphorylated for a longer period of time (Kim et al., 2018). A critical gap in the understanding of control over dynamic oxidation/phosphorylating events is the source, location and duration of ROS governing these processes (Meng et al., 2002; Ostman et al., 2011; Londhe et al., 2020).

**FIGURE 2:**
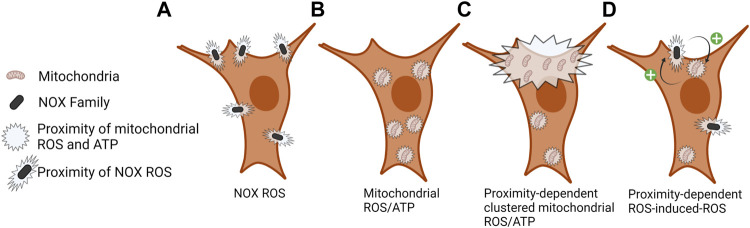
Local proximity of NOX ROS and mitochondrial ROS/ATP. **(A)** ROS is produced by NOX enzymes in the extracellular space proximal to the plasma membrane, signaling at the cytoplasmic face of the plasma membrane is dependent on local ROS concentration and ROS scavenging. **(B)** Mitochondrial ROS (mROS) and ATP are rapidly consumed at the site of production; therefore, the density of these mitochondrial outputs is localized to sites of mitochondrial density. **(C)** Clustering of mitochondria at subcellular sites contributes to a localized increase in ROS and ATP levels. **(D)** ROS-induced-ROS and mitochondrial–NOX crosstalk regulates the activity of each entity and the amount and duration of ROS production. Still unclear is how the proximity of NOX and mitochondria might regulate this process.

ROS can be generated from a variety of sources both externally and internally to the cell. Such internal sources are derived from NADPH oxidases (NOXs) as well as mitochondria via the electron transport chain (ETC). Cellular ROS has also been shown to be produced via the endoplasmic reticulum (ER) (Cao and Kaufman, 2014), peroxisomes (Sandalio et al., 2013), and various enzymatic reactions; however, the main sources of subcellular ROS are derived from the NOXs and mitochondria. The NOX family can be separated into two categories: NOXs and dual oxidases (DUOXs) both of which are membrane bound enzymes that typically extend from the cytosolic face to the extracellular space with ROS generation (superoxide (O_2_^-^) and H_2_O_2_) towards the exterior of the cell (Panday et al., 2015) (Figure 2). ROS generation by these enzymes is regulated by NADPH, protein cofactors, various stimuli, such as bacterial infection, calcium, and post-translation modifications (phosphorylation), to produce ROS, specifically O_2_^−^ and H_2_O_2_ for the NOXs and H_2_O_2_ for the DUOXs (Panday et al., 2015). Subcellular localization of specific NOX isoforms has also been identified with NOX4 being localized to the mitochondria (Shanmugasundaram et al., 2017), nucleus, ER, and directly interacting with focal adhesions (FAs) (Block et al., 2009), as well as NOX2 being localized to the plasma membrane (Anilkumar et al., 2008). FAs are multiprotein segments of a cell responsible for cell attachment by connecting the cytoplasm to the extracellular matrix (ECM). Strategic localization to these subcellular compartments is shown to provide a burst of ROS needed for microbial killing and to inhibit local phosphatases, which contributes to cell migration or increased insulin signaling (Wu et al., 2005; Chen et al., 2008). During FA maturation, NOX4 has been shown to provide the ROS needed for the oxidation of two cysteine residues in actin which is critical in the binding of vinculin, a FA protein that links integrins to the actin cytoskeleton (Vukelic et al., 2018). DUOX specific H_2_O_2_ is also important for epithelial cell migration and rearrangement of the cytoskeleton, which will be discussed later in this review.

NOX enzymes have also been shown to contribute to a gradient of H_2_O_2_ in zebrafish tissues in response to injury. Following tail fin amputations, a 30 µm wide H_2_O_2_ gradient extending from the wound margin into the tissue has been observed (Niethammer et al., 2009; Jelcic et al., 2017). This NOX associated H_2_O_2_ gradient acts as a chemoattractant for inflammatory cell recruitment to aid in repair of the injury. More localized requirements for ROS have also been observed in the repair of the plasma membrane following plasma membrane injury (PMI), which will be discussed more later (Horn et al., 2020). Thus, NOX-dependent ROS gradients on both the micro and macro level contribute to the regulation of cell signaling cascades to aid in repair of tissues.

**FIGURE 3:**
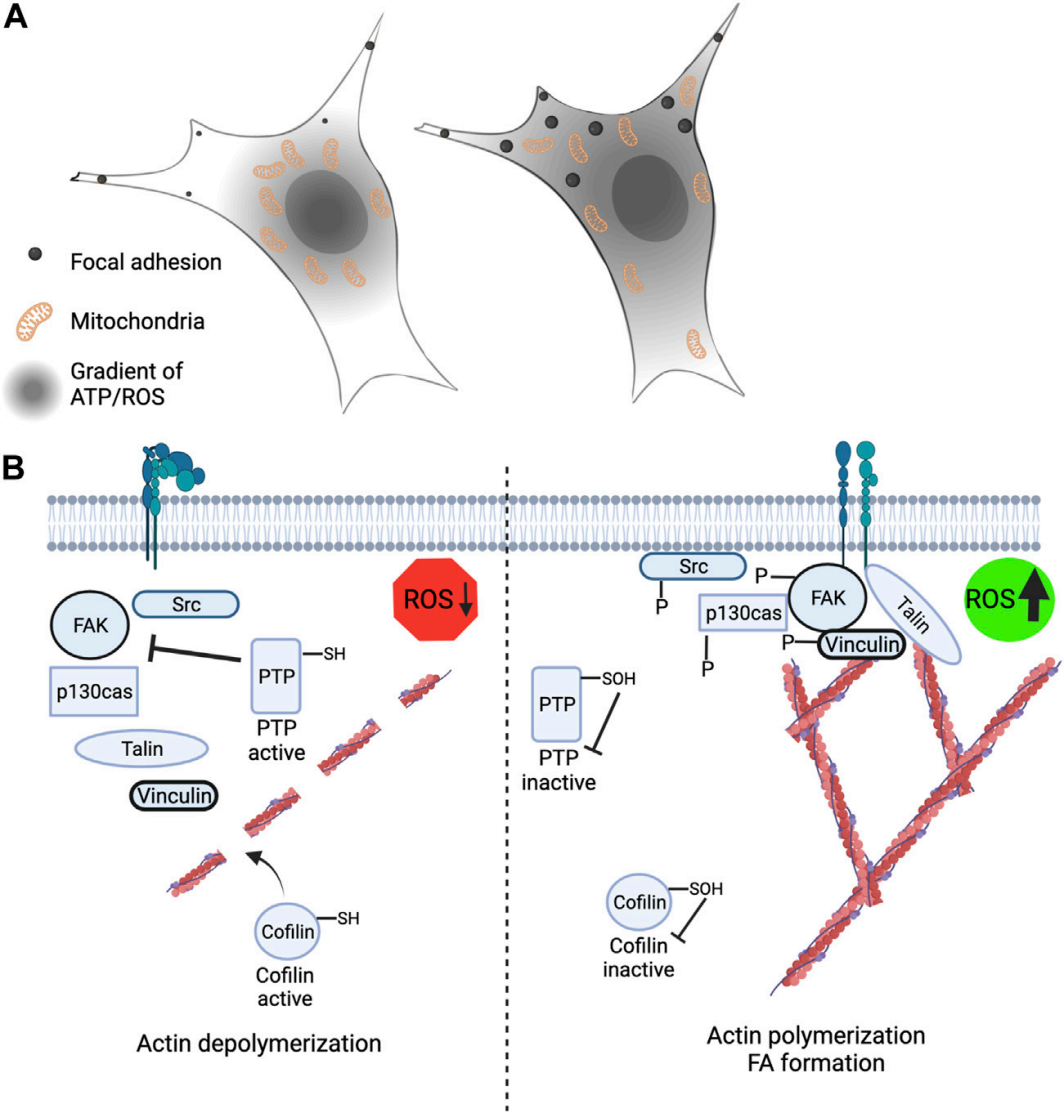
Leading edge mitochondria and redox signaling contribute to cytoskeleton rearrangement and cell migration. **(A)** (Left) Restriction of mitochondria to the perinuclear space leads to loss of peripheral ATP and ROS levels and correlates with smaller and less stable focal adhesions. (Right) Mitochondria that are strategically localized and recruited to the cell periphery have an extended gradient of ATP and mROS and is correlated with larger and more stable focal adhesions. **(B)** (Left) Low ROS levels at the cell periphery promotes actin severing through cofilin activation and increased protein tyrosine phosphatase activity leading to reduced phosphorylation of FAK, p130cas, vinculin and Src. (Right) Elevated ROS levels at the cell periphery promotes actin polymerization and branching through redox-dependent inactivation of cofilin. Inactivation of PTP’s by ROS promotes increased FAK, p130cas, vinculin and Src phosphorylation.

Unlike the NOXs which are membrane bound, the mitochondria are dynamic as they undergo cycles of fission and fusion, as well as are trafficked throughout the cytoplasm (Lopez-Domenech et al., 2018; Horn et al., 2020) (Figures 2, 3). Mitochondria provide a major cellular source of ROS via the ETC (Inoue et al., 2003). mROS are generated in the mitochondrial matrix and IMS by ETC complexes I and III, respectively, as a result of the single electron reduction of O_2_ to produce O_2_^−^ which can be converted to H_2_O_2_ (Murphy, 2009). Manganese Superoxide Dismutase (MnSOD), located in the mitochondrial matrix, catalyzes the reaction of O_2_^−^ to H_2_O_2_, thus changing the type of ROS, but not fully reducing it to H_2_O (Inoue et al., 2003). Complete reduction of H_2_O_2_ to H_2_O in the mitochondrial matrix is accomplished by mitochondrial glutathione peroxidase 4 (GPX4) (Handy et al., 2009) and peroxiredoxin 3 (PRX3) (Newick et al., 2012). Therefore, mROS diffusion out of the mitochondrial matrix will be dependent on the amount of ROS produced in time and space and the activity of resident ROS scavenging enzymes. mROS contribute to redox signaling through canonical cysteine oxidation of target proteins and through retrograde signaling to the nucleus (Tan and Finkel, 2020).

Both ATP and ROS are rapidly consumed at sites proximal to their source, largely due to the abundance of antioxidant enzymes present in the cell (Jones, 2010; Jelcic et al., 2017; Alshaabi et al., 2021) (Figure 2). An additional level of regulation is achieved through the compartmentalization of oxidant and antioxidant systems, allowing cells to utilize redox-dependent systems for physiological signaling and damage responses while protecting redox-sensitive cell compartments (Go and Jones, 2008; Pak et al., 2020). Recent studies in yeast described a mitochondria-to-cytosol H_2_O_2_ gradient where the mitochondrial H_2_O_2_ is rapidly consumed by the cytosolic antioxidant peroxiredoxin, thus the downstream signaling effects of mitochondrial H_2_O_2_ occurs proximal to its site of production (Carmona et al., 2019; de Cubas et al., 2021). Other studies show a strong correlation between mitochondrial matrix H_2_O_2_ levels and cellular growth rate (Morgan et al., 2016). Similar findings have also been described in mammalian cells and the subcellular localization of mitochondria has also been correlated with spatial cytosolic H_2_O_2_ levels (Alshaabi et al., 2021). These collective studies support an inside-out (mitochondrial-cytosolic) redox-signaling gradient from mitochondria.

In support of mitochondria H_2_O_2_ contributing to signaling in cancer cell metastasis, published reports have shown that mitochondria with experimentally decreased ETC function contributed to metastatic phenotypes; results showed an increase in migratory and invasive activity (Porporato et al., 2014). Their findings showed that in their “supermetastatic” and “superinvasive” cell lines there are defects in the TCA cycle characterized by increased succinate production. The unequal pairing of the TCA cycle with the ETC led to an increase in mROS; a notable increase in superoxide production was detected. The increase of succinate and superoxide suggests that more electrons could be transferred to ETC complex II by succinate, resulting in an overloaded ETC. Use of the mitochondria-targeted superoxide scavenger mitoTEMPO resulted in a decrease in tumor cell metastasis (Porporato et al., 2014). This work reinforces the suspected role of mitochondria in cancer aggressiveness and progression. Mutations in mitochondrial DNA (mtDNA) can result in ETC dysfunction, specifically relating to mutations in complex I where both mtDNA and nuclear DNA are required for its formation (Ishikawa et al., 2008). It is important to note that many carcinogenic chemicals are known to bind to mtDNA (Chen et al., 2004; Budnik et al., 2013). It was determined that mtDNA with mutations causing complex I dysfunction increased metastatic phenotypes in transformed cells but did not induce tumor formation in murine models (Ishikawa et al., 2008). Defective complex I function results in ROS accumulation in tumor cells. The mutations to complex I lead to the up-regulation of three genes with heavy implications in metastatic potential: MCL-1, HIF-1α, and VEGF (Ishikawa et al., 2008). The specific role and location of mitochondria in driving these supermetastatic processes is not clear, but likely local mitochondrial recruitment is required (Altieri, 2019).

Although not fully understood, crosstalk between mitochondria and NOX enzymes has been proposed by a mechanism termed “ROS-induced ROS release” (Zorov et al., 2000). ROS-dependent oxidation of mitochondrial ATP-sensitive potassium channels (Queliconi et al., 2011) and full enzymatic activity of NADPH oxidases is required for angiotensin II mediated mROS production (Doughan et al., 2008). Alternatively, mROS have been shown to activate NOX1 following serum withdrawal in human embryonic kidney 293T cells (Lee et al., 2006). Still missing from these studies is the role of subcellular mitochondrial positioning in mediating the initiation and execution of ROS-induced ROS release (Figure 2D). Better understanding the localization and abundance of mitochondria and subcellular ATP and ROS can lead to deciphering local cell signaling cascades in mediating mitochondria and NOX ROS-induced ROS release.

### Mitochondrial trafficking dynamics

In mammalian cells mitochondria are strategically positioned throughout the cytoplasm to meet local energy requirements (Hollenbeck, 2005). This movement is orchestrated by the microtubule motor proteins kinesin and dynein (Fransson et al., 2006; Lopez-Domenech et al., 2018) and allows the mitochondria to move anterograde (to periphery) and retrograde (towards the nucleus), respectively. The actin cytoskeleton and myosin proteins also play a role in mitochondrial trafficking and anchoring, although this is believed to support short movements (Sheng, 2014). The microtubule motor proteins are linked to mitochondria via the TRAK1/2 (Milton) adapter proteins which connect to the outer mitochondrial membrane bound adaptor protein Miro1 or Miro2 (Debattisti et al., 2017; Li et al., 2021). When Miro1 is knocked out from many cell types this results in mitochondria becoming restricted around the nucleus compared to when Miro1 is present, and the mitochondria are strategically and dynamically re-localized throughout the cytoplasm (Ahmad et al., 2014; Schuler et al., 2017; Alshaabi et al., 2021) (Figure 3A). We recently have shown that the subcellular positioning of mitochondria by Miro1 directly impacts intracellular gradients of ATP and mROS (Schuler et al., 2017; Alshaabi et al., 2021) (Figure 3A). Additionally, disruption of the microtubule cytoskeleton with Taxol causes restriction of mitochondria around the nucleus comparable to Miro1 deletion and similar disruption to subcellular H_2_O_2_ gradients. Re-expression of Miro1 can rescue these gradient defects (Alshaabi et al., 2021). Loss of Miro2 does not elicit dramatic changes to mitochondrial trafficking in differentiated cells and therefore has been of less focus (Nguyen et al., 2014).

Another process that impacts mitochondrial trafficking is fission and fusion. To mitigate the effects of damaged mitochondria, a healthy and damaged mitochondrion may fuse together which can be trafficked to areas of the cell in high energy demand (Detmer and Chan, 2007). Mitochondria can also undergo fission which will cause one mitochondrion to split into two and this may support increased trafficking. The role of fission and fusion on mitochondrial trafficking is still unclear; however, fusion can be directly affected by AMP-activated protein kinase (AMPK), a cytoplasmic energy sensor. Therefore, mechanistically providing the cell with information when energy is low in various parts of the cell which in turn signals mitochondria to fragment and be transported to that area (Cunniff et al., 2016; Toyama et al., 2016). Overall, energy sensing plays a role in mitochondrial structure and location.

Mitochondria are also stopped and anchored at specific subcellular sites where mitochondrial functions are required. At sites of high energy demand in neurons mitochondria stop moving, partly by the protein syntaphilin which binds mitochondria to the microtubules (Kang et al., 2008). Mitochondrial movement is also halted in axons at sites of increased calcium (Ca^2+^) (Yi et al., 2004). EF-hands present in the Miro1 protein (Smith et al., 2020) are thought to play a role in this sensing, but there is also evidence that mitochondria can be halted at sites of increased Ca^2+^ when Miro1 is lost (Nguyen et al., 2014). ROS have also been shown to regulate the speed of mitochondrial trafficking, presumably through the p38 MAPK pathway (Debattisti et al., 2017). Mitochondria also respond to increased levels of extracellular glucose, where O-GlcNAc transferase (OGT) performs the glucose-dependent O-GlcNAcylation on key serine residues of the adaptor protein Milton which stops mitochondrial motility (Pekkurnaz et al., 2014). Similarly, by inhibiting the glucose transporter FGT-1 in *Caenorhabditis elegans*, there was a decreased mitochondrial recruitment to the basal membrane to help drive anchor cell (AC) invasion, which is responsible for the development of the reproductive system (Garde et al., 2022).

Another energy dependent process, the activation of AMPK, has been shown to contribute to the recruitment of mitochondria to the leading edge of migrating cells. When AMPK is selectively activated at the leading edge of the cell, mitochondria are trafficked to this specific area accompanied by increased ATP concentration and membrane ruffling, a direct readout of cell migration (Cunniff et al., 2016). Inhibition of mitochondrial activity with acute exposure to the complex I inhibitor, rotenone, blocked membrane ruffling. Local specific and temporal AMPK inactivation, using pharmacological and optogenetic approaches, caused decreased mitochondrial movement to the leading edge as well as decreased cell migration and invasion (Cunniff et al., 2016). These studies provide evidence that when the ATP: ADP ratio is spatially decreased, AMPK becomes activated to drive mitochondria to the site of interest to produce more ATP needed for various downstream signaling at the periphery of the cell. Mitochondrial fission through DRP1 activation is also mediated by AMPK activity in response to ETC inhibition (Toyama et al., 2016). Collectively, numerous metabolic dependent and independent processes converge to mediate the subcellular trafficking, anchoring, and severing of mitochondria to provide local mitochondrial byproducts to areas in demand.

### Leading edge mitochondria can rearrange the cytoskeleton

As described above, subcellular H_2_O_2_ gradients have been shown to regulate cell signaling. Below we will discuss this in the context of phosphorylation dynamics, cytoskeleton remodeling and cell migration. ROS, in particular H_2_O_2_, can act on numerous signaling pathways controlling cell migration including receptor activation, kinase and phosphatase activity, FA dynamics, membrane reorganization and transcription factor activation (Hurd et al., 2012; Truong and Carroll, 2013) (Figure 3). During cell migration and invasion, the mitochondria have been found to localize to the leading edge of the cell to help drive cytoskeleton rearrangements (Madan et al., 2021). Anchor Cell (AC) invasion of the basement membrane (BM) in *C. elegans* requires mitochondrial recruitment to the invasive edge of the AC to drive invadopodia formation (Garde and Sherwood, 2021; Garde et al., 2022). Filamentous actin (F-actin) is responsible for the structure of the invadopodia and is increased by the presence of mitochondria at the invasive edge which provides a local source of ATP (Kelley et al., 2019). Localized ATP at the leading edge of the cell is necessary for the activation of the Arp2/3 complex which serves as a nucleation site for actin filaments. Arp2/3 is activated upon phosphorylation at Thr237/238 in Arp2 and this allows for increased lamellipodia at the leading edge of the cell through the branching of actin filaments (LeClaire et al., 2008). Therefore, the presence of mitochondria at the leading edge of the cell supports increased ATP concentrations to drive protein phosphorylation for the reconstruction of the cytoplasm.

As critical as phosphorylation events, protein oxidation plays a key role in the stability of actin filaments. Oxidation of actin filaments specifically in cell protrusions has recently been described using the ratiometric H_2_O_2_ biosensor HyPer7 fused to the actin binding peptide LifeAct. Using this probe, protrusions with elevated H_2_O_2_ levels were more stable compared to protrusions with lower H_2_O_2_ levels (Pak et al., 2020). This means that mitochondria can serve at least two purposes at the edge of the cell: 1) in providing the ATP needed for Arp2/3 activation for F-actin formation 2) in providing sufficient ROS needed to maintain F-actin stability. Similarly, mitochondria are required at the site of plasma membrane injury (PMI) to provide the necessary means for plasma membrane repair (PMR). At the site of PMI in mouse embryonic fibroblasts (MEFs), mitochondria fragment and this supports signaling to aid in repair, cells that lack the required machinery for mitochondrial fission (DRP1) fail to repair (Horn et al., 2020). The small GTPase, DRP1, oligomerizes around the mitochondrial outer membrane and is necessary for pinching of one mitochondrion into two via fission (Rosdah et al., 2020). The DRP1 adaptor protein MiD49 is involved in mitochondrial fission and when this is absent from the cell they fail to repair, and the mitochondria are not able to sustain increased calcium intake at the site of injury (Horn et al., 2020). Fragmented mitochondria cause an increase in F-actin abundance at the site of injury which aids in repairing the plasma membrane; however, unfragmented mitochondria fail to effectively heal the plasma membrane. Localized mROS production also contribute to plasma membrane repair through activation of RhoA and actin polymerization (Horn et al., 2017). These DRP1-dependent responses only occur proximal to the site of membrane damage. DRP1 is also upregulated in many cancer cells, including metastatic breast cancer cells (Zhao et al., 2013). DRP1-dependnet fission is thought to support fragmentation of mitochondria for subcellular transport (Giovarelli et al., 2020). Silencing of DRP1 in breast cancer cells decreases mitochondrial fission, cell migration and invasion (Zhao et al., 2013). Loss of DRP1 also accompanied a reduction in the number of mitochondria in the leading edge of these cells. DRP1 also supports the directional migration of breast cancer cells, supporting the movement of mitochondria to the anterior membrane in the direction of cell migration (Desai et al., 2013). Thus, mitochondrial fission and location are important in F-actin dynamics and cell migration.

Cell migration and invasion *in vivo* requires degradation and remodeling of the extracellular matrix (Bonnans et al., 2014). The primary set of enzymes known to degrade the extracellular matrix are the matrix metalloproteinases (MMPs) (Loffek et al., 2011). MMPs are also regulated via reversible oxidation and phosphorylation. Increasing intracellular H_2_O_2_ levels via MnSOD, the mitochondrial superoxide dismutase, as well as increasing mROS via rotenone and antimycin A increases the activity of the MMPs (Hazan et al., 2000). The expression levels of MMP-1 is increased by intracellular ROS concentrations; therefore, both the activity and expression levels are increased in the presence of elevated ROS (Shin et al., 2015). Cell migration and invasion are also correlated with the activity of MMPs in breast cancer cells (Ren et al., 2015). Oxidation activates MMPs; however, phosphorylation inactivates them, and it is believed that protein kinase C (PKC) is the kinase responsible for their inactivation (Sariahmetoglu et al., 2007; Williams and Coppolino, 2011). It is not fully understood if oxidation or phosphorylation is dominant when both species are present, thus these two post translational modifications do not crosstalk with each other, per se, but they do have opposing functions on MMPs. Therefore, mitochondria are critical in the regulation of MMPs which influence cell migration and invasion via reshaping the extracellular milieu.

### mROS can alter localized phosphorylation status

The relationship between the positioning of mitochondria within the cell and the downstream effects on cell migration, invasion, and membrane repair are starting to be revealed; however, these processes are not fully understood (Cunniff et al., 2016; Schuler et al., 2017; Horn et al., 2020; Garde and Sherwood, 2021). Mitochondrial positioning directly maps to area of increased ATP as well as H_2_O_2_ which makes intracellular trafficking of these organelles critical for the function of the cell. When mitochondria are concentrated to the perinuclear area this causes a decrease in ATP and H_2_O_2_ concentrations in the cell periphery and an increased in perinuclear H_2_O_2_ levels; however, this is rescued when mitochondrial trafficking to the periphery is rescued (Schuler et al., 2017; Alshaabi et al., 2021).

Few relationships between mitochondrial positioning and the effects of their byproducts, ATP and H_2_O_2_, on proteins have yet to be fully understood. Two targets to have altered function based on Miro1-mediated mitochondrial positioning are vinculin, which is a cytoplasmic protein involved in the binding of actin in focal adhesions, and p130cas, which serves as a substrate for several tyrosine kinases (Peng et al., 2011) (Figure 3B). When mitochondria are restricted around the nucleus due to deletion of Miro1 (Miro1^−/−^) in MEFs there is decreased H_2_O_2_ in the cell periphery (Alshaabi et al., 2021) (Figure 3A). This correlates with lower vinculin and p130cas phosphorylation at tyrosine residues Y100 and Y410, respectively, residues critical for activity (Pellicena and Miller, 2001; Golji et al., 2012). When Miro1 is re-expressed via stable expression of Myc-tagged Miro1 in MEFs the mitochondria are redistributed throughout the cytoplasm causing an increase in H_2_O_2_ in the periphery, accompanied by increased phosphorylation of vinculin and p130cas (Alshaabi et al., 2021). Going alongside this, it has been shown that elevated H_2_O_2_ levels in metastatic bladder cancer cells increases the phosphorylation and membrane recruitment of p130cas through oxidation of the PTPN12 phosphatase, driving the metastatic phenotype (Hempel et al., 2013). Mitochondrial and NOX-dependent sources of ROS have both been implicated in regulation of these processes stated above, but due to the intimate crosstalk between mitochondria and NOX enzymes (Daiber, 2010), deciphering the precise contribution from each source has been challenging (Figure 2D).

Similarly, during cell migration, there is an increase in ROS in cell protrusions which is needed for the oxidation of cofilin at C139 and C147 (Cameron et al., 2015). Cofilin is a cytoplasmic protein that is responsible for the severing of F-actin. When oxidized at C139 and C147 cofilin becomes inactivated (Figure 3B). Oxidation resistant mutants of cofilin were shown to reduce breast cancer attachment, migration, and invasion (Cameron et al., 2015). Cofilin is also regulated via phosphorylation and when phosphorylated at S3 it renders the protein inactive (Agnew et al., 1995; Moriyama et al., 1996; Sumi et al., 1999). Since ATP and H_2_O_2_ are abundant in areas of high mitochondrial density (Schuler et al., 2017; Alshaabi et al., 2021) it is realistic that either or both molecules could regulate cofilin activity, however, it is unclear which molecule is preferentially utilized from mitochondria.

All the proteins listed above: vinculin, p130cas, and cofilin are all important in FA formation which aids in cell attachment and migration. Focal adhesion kinase (FAK) is a key kinase found in FA formations and it is known to be activated via phosphorylation; however, its phosphorylation is attenuated by inhibition of redox signaling in the cell periphery (Chiarugi et al., 2003) (Figure 3B). FAK dephosphorylation/inactivation can be positively regulated via integrin-induced ROS which inhibits low molecular weight protein tyrosine phosphatase (LMW-PTP), therefore keeping FAK activated for longer (Chiarugi et al., 2003; Scales and Parsons, 2011). Subcellular ROS has also been shown to activate FA proteins such as FAK, paxillin, and p130cas, which all are integral in FA maturation and cell adhesion (Gozin et al., 1998). Disruptions in the trafficking of mitochondria and changes in local H_2_O_2_ and ATP levels correlate with perturbations in FA dynamics (Schuler et al., 2017). Leading edge changes in mROS also contribute to Src and FAK signaling driving breast cancer cell migration. Downregulation of SIRT3 in breast cancer cells supports increased mROS signaling that increases Src-dependent phosphorylation of FAK (Tyr576/577) and p130Cas (Y410) at the leading-edge membrane (Lee et al., 2018). SIRT3 mediated changes in Src and FAK phosphorylation were also sensitive to addition of endogenous antioxidants. While performing scratch-migration assays, it was observed that SIRT3 levels were the lowest in cells at the leading edge of the scratch, compared to non-migrating cells at distal sites, indicating migrating cells downregulate SIRT3 expression to support increased mROS mediating Src and FAK phosphorylation (Lee et al., 2018).

FAK and Src activities are closely intertwined since they participate in overlapping signaling response. Oxidative stress, elicited by PI3 kinase, in Caco-2 colon epithelial cells, caused increased activity and phosphorylation of FAK at Y397, Y577, and Y925 as well as c-Src activity and phosphorylation at Y418 (Basuroy et al., 2010). This resulted in increased cell migration, but by expressing a dominant negative c-Src the oxidant induced cell migration was prevented; therefore, it was found that both oxidants and an active c-Src were needed to rapidly increase cell migration via FAK (Basuroy et al., 2010). Similarly in vascular endothelial cells, FAK is activated and phosphorylated in the presence of H_2_O_2_ in a time and dose dependent manner (Vepa et al., 1999). The increased FAK activity also corresponded with enhanced actin stress fibers because of cytoskeleton reorganization.

During cell attachment there is an integrin-induced release of ROS at the plasma membrane which oxidizes Src, therefore increasing Src activity by dephosphorylating Y527. Src activity has been linked to increased cell invasion and tumor onset; however, when antioxidants are used or an oxidant null Src (C245A and C487A) is expressed then Src activity decreases as well as cell invasion and tumor progression (Giannoni et al., 2005) (Figure 3B). Oxidation of Src via ATP-mediated activation of DUOX1-dependent H_2_O_2_ production increases Src activity which activates the epidermal growth factor receptor (EGFR) to activate downstream signaling pathways (Truong and Carroll, 2012; Heppner et al., 2016). DUOX-1 activity is also important for epithelial cell migration during repair via activation of EGFR (Gorissen et al., 2013). Still unclear is the role of mitochondria is these processes which presumably is important given the regulation of DUOX enzymes by ATP and Ca^2+^.

### Gap in knowledge/Summary

The regulation of redox-dependent signaling by mitochondrial or NOX-dependent ROS production is well-established and new targets are continuously being uncovered. The crosstalk between these ROS sources, with distinct differences in subcellular localization, dynamics, substrates, and targets is still unclear. The dynamic nature of the mitochondria and the ability to produce both ATP and ROS at specific subcellular sites provides an additional layer of control to redox and phospho-signaling by mitochondria. The contribution of local mitochondrial populations and how disruption of the subcellular architecture of mitochondria may impact NOX activity is unclear. We hypothesize that disruption of intracellular ATP and ROS gradients via loss of Miro1 mediated mitochondrial positioning, or other mitochondrial disruptions, would alter NOX-dependent redox signaling and redox-dependent phosphorylation cascades. Critical gaps still exist regarding the role of localized mitochondria in regulating these signaling events during cell migration and other localized responses (ie. membrane repair). Herein, we have briefly summarized the literature that supports the subcellular trafficking of mitochondria in the regulation of redox and phospho-signaling events supporting cell migration, linking mitochondrial dynamics to the spatial and temporal control over redox and phospho-signaling cascades.
